# High-throughput ab-initio dilute solute diffusion database

**DOI:** 10.1038/sdata.2016.54

**Published:** 2016-07-19

**Authors:** Henry Wu, Tam Mayeshiba, Dane Morgan

**Affiliations:** 1 Department of Materials Science and Engineering, University of Wisconsin-Madison, Madison, Wisconsin 53706, USA; 2 Materials Science Program, University of Wisconsin-Madison, Madison, Wisconsin 53706, USA

**Keywords:** Density functional theory, Computational methods, Atomistic models

## Abstract

We demonstrate automated generation of diffusion databases from high-throughput density functional theory (DFT) calculations. A total of more than 230 dilute solute diffusion systems in Mg, Al, Cu, Ni, Pd, and Pt host lattices have been determined using multi-frequency diffusion models. We apply a correction method for solute diffusion in alloys using experimental and simulated values of host self-diffusivity. We find good agreement with experimental solute diffusion data, obtaining a weighted activation barrier RMS error of 0.176 eV when excluding magnetic solutes in non-magnetic alloys. The compiled database is the largest collection of consistently calculated *ab-initio* solute diffusion data in the world.

## Background & Summary

Solute diffusion is the way in which impurities are transported in alloys, and many important material properties depend critically upon this transport, such as phase transition kinetics^1–3^. In general solute diffusion is controlled by the random jumps of point defects within the material. In the case of vacancy mediated diffusion in dilute solid solution alloys, the impurity diffusion coefficient can be accurately predicted from the rates of atomic vacancy exchanges around the impurity, and robust formulae have been developed for major crystal structures^4^.

Despite the importance of impurity diffusion coefficients, only a small fraction of dilute binary alloy diffusivities have been experimentally measured^5,6^. The limited data is due to many experimental challenges, including a lack of corresponding radioactive tracer, detection limitations for slow diffusers, and metastability of the host crystal structure, as well as simply the time and cost of exploring the tens of thousands of possible systems. First-principles theoretical methods overcome these issues, as they are able to utilize a wide variety of elemental species, sample and quantify high activation barriers, work with metastable crystal structures, and can be performed relatively cheaply and quickly compared to experiments when properly automated. A computational approach is also able to provide the diffusion data in a consistent framework, allowing all diffusivities to be compared on equal footing.

Expanding upon previous theoretical studies of dilute solute diffusion in alloys^7–14^, we present in this work the largest consistently calculated *ab-initio* solute diffusion database to-date. This database consists of more than 230 dilute solute diffusion systems in Mg, Al, Cu, Ni, Pd, and Pt hosts. These diffusion calculations were automated using our high-throughput workflow software, the MAterials Simulation Toolkit (MAST),^15,16^ developed at the University of Wisconsin-Madison. MAST is built upon pymatgen^17^ and automatically handles input/output processing of *ab-initio* calculations and manages job submission to cluster queues. MAST can be used to control complex workflows, and was used here to manage multifrequency model calculations on a large number of systems.

The paper is organized as follows. We first briefly outline our computational methodology for generating dilute solute diffusion data and detail our empirical corrections. An overview of the structure and description of the data will then be presented. Finally we demonstrate the validity of our data with an analysis of associated DFT errors and comparisons to experimental diffusion measurements.

## Methods

### Computational methods

We perform all calculations using the Vienna *ab-initio* Simulation Package (VASP)^18–21^ version 5.3.3. We treat exchange–correlation in the Generalized Gradient Approximation (GGA), as parameterized by Perdew, Burke, and Ernzerhof (PBE)^22,23^. The projector augmented wave method (PAW)^24,25^ pseudopotentials were used with a plane wave cutoff of 350 eV for all systems. The constant 350 eV energy cutoff was used to keep consistency and is higher than the largest ENMAX of elements calculated. Bulk and defect calculations were done using 4×4×3 HCP conventional supercells for Mg alloys containing 96 atoms and 3×3×3 cubic FCC supercells for Al, Cu, Ni, Pd, and Pt alloys containing 108 atoms. The Brillouin zone was sampled by a 5×5×5 Gamma centered mesh for the HCP supercells and a 4×4×4 Monkhorst-Pack k-point mesh for the FCC supercells. Errors in energy are converged to less than 1 meV/atom with respect to the energy cutoff and k-points; errors in force are relaxed to less than 0.01 eV/Å. All runs that require magnetization were done as spin-polarized calculations; these include all Ni alloys, and Cr, Mn, Fe, Co, and Ni solutes. The need to run spin-polarized calculations for magnetic solutes in non-magnetic hosts has previously^8,11^ been found to be essential for diffusion calculations. Additional computational method effects such as finite supercell errors and comparison between different exchange-correlation functionals will be discussed in the validation section.

Migration barriers for atomic jumps were calculated using the climbing image nudged elastic band (CI-NEB) method with a single intermediate image. For the transitions we consider, which are single atom jumps to nearest neighbor sites, a single image is sufficient to determine the transition saddle point. Migration attempt frequencies (*υ*_*hop*_) were calculated with the Vineyard^26^ approach. However, rather than computing all 3*n* vibrational modes, we consider only the vibrational modes of the hopping atom (with all other atoms held fixed) in its initial position (*υ*^*initial*^) and at the saddle point configuration (*υ*^*saddle*^):

$$\nu_{hop}=\frac{\prod_{1}^{3n}\nu_{i}^{initial}}{\prod_{1}^{3n-1}\nu_{i}^{saddle}}∼\frac{\prod_{1}^{3}\nu_{i}^{initial}}{\prod_{1}^{2}\nu_{i}^{saddle}}.$$

### Dilute solute diffusion models

**Figure 1:**
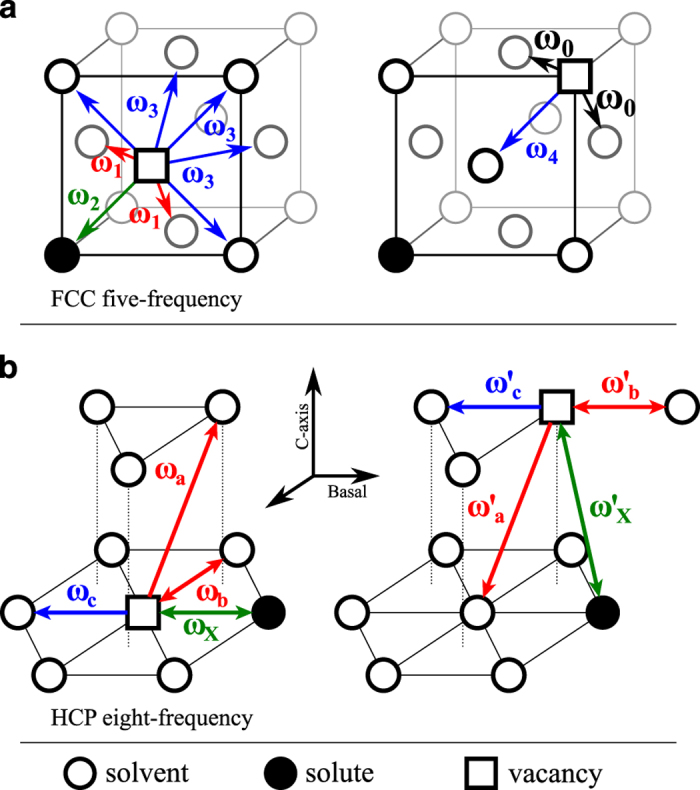
Schematic diagram depicting the atomic hops considered in our calculations, for (**a**) the FCC five-frequency diffusion model, and (**b**) the HCP eight-frequency diffusion model.

We calculate solute diffusion coefficients by following the multi-frequency framework developed by LeClaire^27^. Figure 1 shows all the atomic jumps we consider for both FCC and HCP hosts. For FCC we use the five-frequency diffusion model^1,4^ (Fig. 1a) and for HCP we use the eight-frequency diffusion model^28^ (Fig. 1b). These diffusion models assume dilute solute concentrations and therefore do not include solute-solute interactions. Each jump frequency (*ω*_*i*_), is calculated from DFT migration barriers (*E*_*i*_) and attempt frequencies (*υ*_*i*_) in the simple Arrhenius expression

$$\omega_{i}=\nu_{\mathrm{i\backslash}}\exp\left(\frac{-E_{i}}{k_{B}T}\right),$$

where *k*_*B*_ is the Boltzmann constant and *T* is the temperature. In the five-frequency FCC diffusion model, *ω*_*0*_ is the bulk vacancy hop rate away from any solutes, *ω*_*1*_ is the vacancy-solute rotation hop, *ω*_*2*_ is the vacancy-solute exchange hop, and *ω*_*3*_ and *ω*_*4*_ are the vacancy-solute dissociation and association hops, respectively. In the eight-frequency HCP diffusion model, *ω*_*a*_ and *ω’*_*a*_ are the vacancy-solute rotation hops from basal orientation to c-axis and vice versa, *ω*_*b*_ and *ω’*_*b*_ are the vacancy-solute rotation hops within the basal and c-axis planes, *ω*_*c*_ and *ω’*_*c*_ are the vacancy-solute dissociation hops from the basal and c-axis configurations, and *ω*_*X*_ and *ω’*_*X*_ are the vacancy-solute exchange hops within the basal and c-axis planes. For the FCC systems, the prefactors for all five frequencies were calculated and included. For the HCP systems, two prefactors were calculated and used, one for all solute atom transitions (*ω*_*X*_ and *ω’*_*X*_) and one for all solvent atom transitions (*ω*_*a*_, *ω’*_*a*_, *ω*_*b*_, *ω’*_*b*_, *ω*_*c*_, and *ω’*_*c*_).

To improve the predictive capabilities of DFT diffusion, we propose a correction on top of direct DFT calculated solute diffusivity, by scaling according to how much the DFT host self-diffusivity deviates from the experimental self-diffusivity. We accomplish this by multiplying the raw DFT diffusivities by a correcting Arrhenius equation,

$$D_{corrected}^{solute}=A_{shif\mathrm{t\backslash}}\exp\left(\frac{-E_{shift}}{k_{B}T}\right)\cdot D_{DFT}^{solute},$$

where the correctional shift parameters, *A*_*shift*_ and *E*_*shift*_, are determined by fitting the DFT host self-diffusivity to experimental measured self diffusivity such that,

$$D_{experiment}^{host}∼A_{shif\mathrm{t\backslash}}\exp\left(\frac{-E_{shift}}{k_{B}T}\right)\cdot D_{DFT}^{host}.$$

Table 1 reports these correction parameters for all six host elements along with the uncorrected and corrected diffusion constants and activation barriers. Because the shift parameters were determined from an Arrhenius fit to all combined experimental data, the corrected diffusion constant and activation barrier essentially represents the average experimental self-diffusivity. The correct diffusion constant results from a direct product between *A*_*shift*_ and the uncorrected diffusion constant. The corrected activation barrier results from a direct summation between *E*_*shift*_ and the uncorrected activation barrier. All solute diffusivities and diffusion parameters reported henceforth will be values after applying this corrective procedure. This correction is not essential but improves results compared to experiments and creates almost no loss of generality for our approach because self-diffusion coefficients are known for almost all the elements of interest.

### Code availability

The MAterials Simulation Toolkit (MAST)^15,16^ is the code package used for the calculation of these diffusion coefficients. MAST is an open-source code released with the Massachusetts Institute of Technology (MIT) license and the latest version is freely accessible at https://pypi.python.org/pypi/MAST. The input files used in this work can be found with MAST version 1.3.3 at the following Zenodo link^16^ http://dx.doi.org/10.5281/zenodo.48656.

## Data Records

The full diffusion dataset is publically available at Figshare (Data Citation 1) and at our own interactive web page (http://diffusiondata.materialshub.org). The data for each host element catalogs the various properties of the host element, hopping properties of the solute in the host, and extracted solute diffusion parameters. There is only one set of host element properties, while additional data columns are used for each additional solute element. The solute diffusion parameters, solute diffusion constant, *D*_*0*_ and solute diffusion activation energy, *Q*, can be used in the following Arrhenius diffusion equation to generate the temperature, *T*, dependent solute diffusivity:

$$D^{solute}\left(T\right)=D_{0}^{solute}\exp\left(\frac{-Q^{solute}}{k_{B}T}\right).$$

### Graphical representation of the results

**Figure 2:**
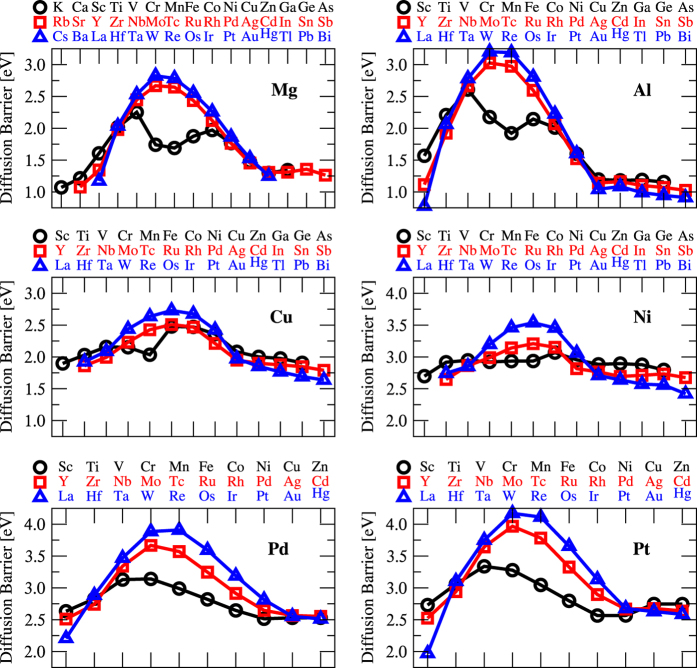
Trend in solute diffusion activation barriers in all host alloys, Mg, Al, Cu, Ni, Pd, and Pt from DFT calculations across the periodic table. The barriers are extracted from the temperature range between the host element’s melting temperature and half melting temperature. For Mg, only the basal diffusion barrier is plotted; the trend for the c-axis diffusion barrier is almost the same.

In Figure 2 we plot the DFT diffusion activation energies in each of the six host alloys. These diffusion activation barriers are extracted from our DFT diffusivities in the temperature range between the host element’s melting temperature and half melting temperature. Quantitative similarities can be seen between the 3d, 4d, and 5d solutes, with a noticeable dip for the 3d magnetic elements, Cr, Mn, Fe, Co, and Ni. While the hosts Mg, Al, or Cu does not show any magnetization; the presence of some of these magnetic solutes induces a moment at the transition state of the solute-vacancy exchange. This effect reduces the energy barrier for those transitions, resulting in the dips seen in Fig. 2. If these solutes were calculated without spin-polarization, the 3d curves would instead follow the same trend as the 4d and 5d curves.

An increase in the diffusion activation energy correlates with an increased d-shell filling, peaking near half d-filling, and then finally decreasing back down as the d-shell completely fills. This smooth change is only broken by the above-mentioned magnetic 3d solutes. The amount of change in the activation energy becomes more significant at higher d-shells, with larger barrier changes in 5d as compared with 3d when moving across the table. Between different d-shells, diffusivities converge and cross over near the Ti/V groups on the left and near the Ni/Cu groups on the right. These transition points are not surprising as elements in these periodic groups are quite similar chemically. The resulting effect gives higher activation energies with higher d-shell within the range between the Ti/V and Ni/Cu groups, and lower activation energy with higher d-shells outside of this range.

## Technical Validation

### Validation with experimental diffusion measurements

**Figure 3:**
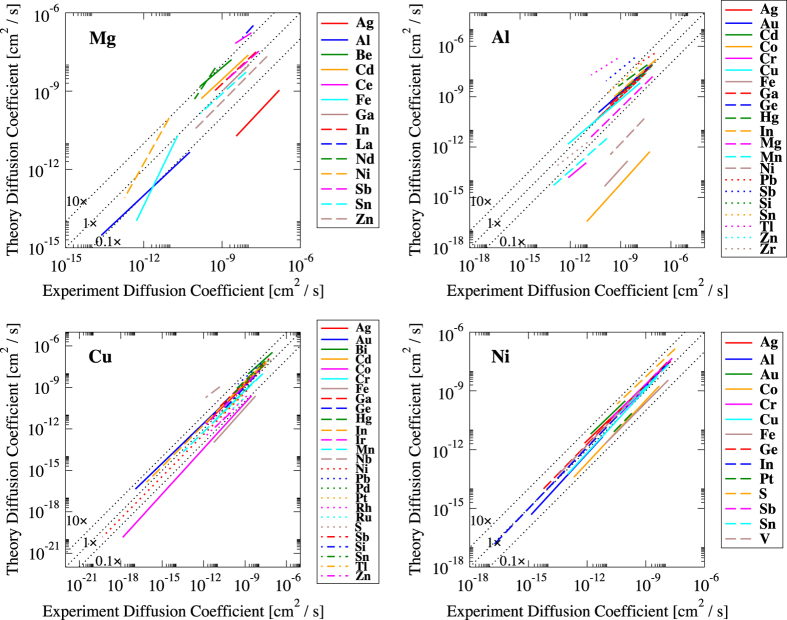
Comparison between DFT solute diffusivities and experimental measurements. Each line represents a solute in Mg, Al, Cu, or Ni. The DFT diffusivity for each solute is matched up with the experimental diffusivity within the experimental temperature range. Dotted black lines represent the identity line (1×) along with 10× and 0.1× DFT versus experiment.

Figure 3 compares corrected DFT diffusion values to experimentally measured diffusion coefficients for dozens of dilute solutes in Mg, Al, Cu, and Ni. In these plots, the DFT diffusivity is shown for the same temperature range as used in the experimental data. Both experimental and DFT values are determined from Arrhenius fits (Eq. (1)) to the exact measurements and calculations. The experimental and DFT values for a given system and temperature are then viewed as an (x,y) pair and plotted. We connect these points with lines since Arrhenius expression trends are perfectly linear on log-log plots. Perfect agreement would result in a 45° y=x line, right along the diagonal. A line that is shifted by a constant off the central diagonal represents a multiplicative factor between theory and experiment, i.e., a discrepancy in $D_{0}^{solute}$ in Eq. (1). Lines that are not on a 45° slope indicate activation barrier differences between theory and experiment, i.e., a discrepancy in *Q*^*solute*^ in Eq. (1). More than half of all solutes in Al and almost all solutes in Mg, Cu, and Ni fall within a factor of 10 with respect to the experiment. The largest diffusivity disagreement is seen for solute diffusion in Al, where DFT over-predicts Tl diffusion by three orders of magnitude and under-predicts Co and Fe diffusion by four orders of magnitude each. In Mg, the solute Ag is under-predicted by DFT, while the largest barrier disagreement is found for Fe and Ni. It is clear that most of the solutes that show large disagreement between theory and experiment are the magnetic elements, Cr, Mn, Fe, Co, and Ni. The close agreement we find for all solutes in Ni, which were all done spin-polarized, suggests that this is not an intrinsic failure for all magnetic calculations. We instead conclude that the issue lies with the configuration of a single solute magnetic moment surrounded by host atoms with no moments. Either DFT is not able to capture all the effects of this interaction, or some other diffusive mechanism is activated by this single atom moment.

We quantify the DFT/experimental agreement using three host-dependent metrics: two solute diffusion barrier RMS errors, for both weighted and unweighted averages, and a solute diffusion coefficient ratio.

The unweighted diffusion barrier RMS error is calculated as:

$$E_{host}^{RMS}[eV]=\sqrt{\frac{\sum_{i=1}^{n}\left[{\left(E_{i}^{DFT}-E_{i}^{expt}\right)}^{2}\right]}{n}},$$

while the weighted diffusion barrier RMS error is computed as:

$$E_{host}^{w-RMS}[eV]=\sqrt{\frac{\sum_{i=1}^{n}\left[\left(\frac{1}{T_{i}^{low}}-\frac{1}{T_{i}^{high}}\right)\cdot {\left(E_{i}^{DFT}-E_{i}^{expt}\right)}^{2}\right]}{\sum_{i=1}^{n}\left(\frac{1}{T_{i}^{low}}-\frac{1}{T_{i}^{high}}\right)}},$$

where $E_{i}^{DFT}$ and $E_{i}^{expt}$ are the DFT and experimental diffusion barriers for solute *i*, respectively, while $T_{i}^{low}$ and $T_{i}^{high}$ form the experimental temperature range in Kelvin for solute *i*, and *n* is the number of solutes compared. This method places lower weights for narrower experimental temperature ranges due to the intrinsically higher fitting error on the experimental diffusion. $E_{host}^{RMS}$ and $E_{host}^{w-RMS}$ represent the diffusion activation barrier RMS error in units of eV for a particular host system, unweighted and weighted, respectively.

The diffusion coefficient ratio metric is the average of the log of ratios of DFT to experimental *D* values, which is computed in the following manner:

$$\log_{10}\left[D_{host}^{ratio}\right]=\frac{\sum_{i=1}^{n}\left|\log_{10}\left[D_{i}^{DFT}/D_{i}^{expt}\right]\right|}{n},$$

where $D_{i}^{DFT}$ and $D_{i}^{expt}$ are average DFT and experimental diffusion coefficients for solute *i*, over the experimental measurement range. $D_{host}^{ratio}$ represents an average deviation factor between DFT and experiment for a particular host system. Please note that the number given is not for the log deviation error, rather it is a direct diffusion ratio factor $D_{host}^{ratio}$. From Fig. 3 we find this metric triplet, ($E_{host}^{RMS}$, $E_{host}^{w-RMS}$, $D_{host}^{ratio}$), to be: (0.404 eV, 0.436 eV, 5.44) for Mg-host, (0.294 eV, 0.229 eV, 14.7) for Al-host, (0.183 eV, 0.134, 3.32) for Cu-host, and (0.130 eV, 0.134 eV 2.30) for Ni-host. Combining all experimental comparisons for these four hosts, we find our performance metric, ($E_{host}^{RMS}$, $E_{host}^{w-RMS}$, $D_{host}^{ratio}$), to be: (0.264 eV, 0.231 eV, 5.16). Excluding the magnetic solutes from non-magnetic hosts, our performance metric improves to: (0.225 eV, 0.176 eV, 3.31).

### Analysis of associated computational errors

To quantify the limitations of our computational methodology, we compute the errors resulting from several aspects of our calculation settings. These include finite-size supercell effects, choice of the exchange-correlation functional, effect of extended solute-vacancy binding, and approximation of the hopping atom attempt frequency.

DFT calculations are widely used because of their efficiency, reliability and transferability. However, they are still generally limited to calculations of less than about 1000 atoms, and typically many fewer for studies involving thousands of calculations. The small periodic supercell sizes can introduce significant finite size cell effects due to strain and other fictitious image effects, and must be carefully considered. We estimate the magnitude of this effect by calculating the vacancy formation and migration energy for Mg with 3×3×2 (36 atoms), 4×4×3 (96 atoms), and 6×6×4 (288 atoms) HCP supercells, and for Pd/Pt with 2×2×2 (32 atoms), 3×3×3 (108 atoms), and 4×4×4 (256 atoms) FCC supercells. We then fit a linear relation between these energies versus the inverse of the total number of atoms at each size. We find that Mg vacancy formation energy is almost independent with respect to system size, while both Pd and Pt vacancy formation energies decrease with system size. The extrapolated formation energy at infinite size, corresponding to the y-intercept of the fit, is within 50 meV of that from the size we use for all future diffusion calculations (4×4×3 for HCP, and 3×3×3 for FCC). The extrapolated vacancy migration energy at infinite size is within 30 meV to that from the size we use. For the smallest Mg supercell size, 3×3×2, we find that only two unit cells in the c-axis direction is clearly insufficient, as the c-axis vacancy migration energy deviates significantly from linear scaling.

In Kohn-Sham DFT, the exchange-correlation (xc) functional is an approximation to the exact exchange interaction and electronic correlation between many-body electrons. Approximating the xc functional is necessary because the exact functional form is unknown. No current xc functional is accurate for all system properties, and a variety of functionals should be tested for the application of interest. We test the self-diffusivity of the six host elements against experimental measurements for four different xc functionals: local density approximation (LDA), Perdue-Wang’91 (PW91), Perdew-Burke-Ernzerhof (PBE), and PBE solid (PBEsol). All of these are widely used exchange-correlation functionals in DFT.

**Figure 4:**
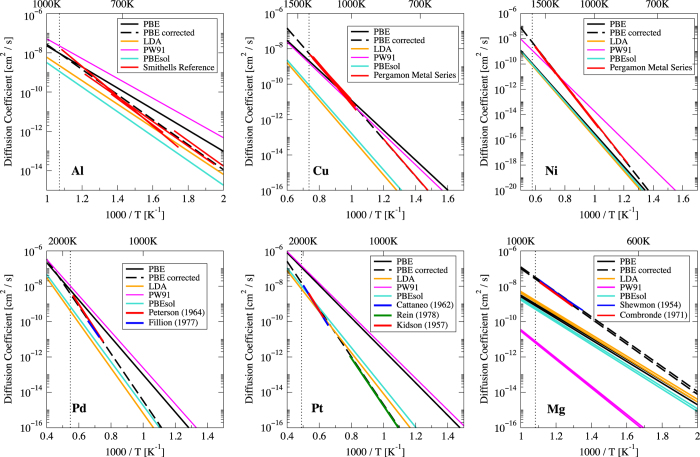
Comparison of self-diffusion predictions from different DFT exchange-correlation functionals (PBE, LDA, PW92, and PBEsol) and the experimentally measured self-diffusion for Al, Cu, Ni, Pd, Pt, and Mg. The dashed black lines on each plot represent the corrected PBE predictions, which have been fitted to the experimental diffusion values. The vertical dotted line on each plot indicates the melting point for the respective host element.

Figure 4 shows the self-diffusivity predictions from the four xc functional as well as the experimentally measured diffusivities for Al, Cu, Ni, Pd, Pt, and Mg. From the data, there is no clear functional which perform significantly better than others. For Cu, the experimental self-diffusion match closely to PBE and PW91, while for Pt, the experiments match more closely to LDA and PBEsol. For Al and Pd, the experimental self-diffusion lies directly in the middle of all four functionals, while for Mg all four functionals under predicts the experiments.

In Table 2, we show the predictions of the vacancy formation and migration energies from PBE, LDA, PW91, and PBEsol for Al, Cu, Ni, Pd, Pt, and Mg. Summing up the vacancy formation and migration energies results in the self-diffusion activation barrier, which are slopes from the lines on Fig. 4. The average xc error shows that unlike the self-diffusivity comparisons, LDA and PBEsol matches better to both experimental vacancy formation and migration energies than PBE and PW91. However deviations are still on the order of several hundreds of meV, with the vacancy formation energy being the being the dominant error. This, coupled with the self-diffusion deviations from Fig. 4 both suggest that self-diffusion corrections would still be required no matter which exchange-correlation functional is used. Since the self-diffusion correction is a direct fit to experimental measurements and with *E*_*shift*_ correcting for mainly the vacancy formation energy, there is likely little difference in choosing between each of these xc functionals, and we have chosen to use the PBE xc functional for all our solute diffusion calculations.

Within the five-frequency model, *ω*_*3*_ and *ω*_*4*_ represent the dissociation and association hops between a solute and vacancy, respectively. This diffusion model assumes only first nearest-neighbor (1NN) interactions between the solute and vacancy, meaning that all energy changes for vacancy movement away from the 1NN configuration are equivalent, whether it be to the second (2NN), third (3NN), or fourth (4NN) nearest-neighbor. The assumed complete dissociation beyond 1NN also allows the difference in energy barrier between *ω*_3_ and *ω*_4_ to act as the solute-vacancy binding energy within the diffusion model. However, since the solute-vacancy interactions in real systems do not stop at 1NN, the magnitude of further neighbor binding and their effect on solute diffusion must be considered.

**Figure 5:**
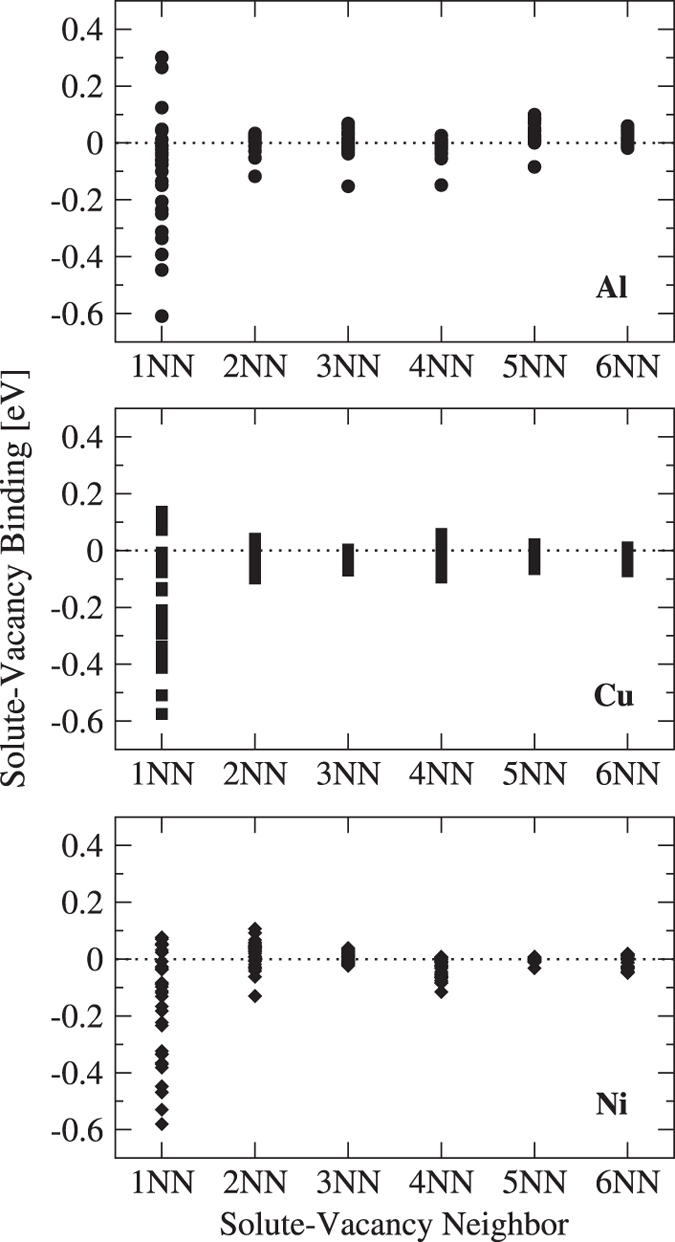
Solute-vacancy binding within Al, Cu, and Ni with respect to neighboring distance. A negative binding indicates an attractive solute-vacancy interaction, and a positive binding indicates a repulsive interaction. Each point represents one solute.

Figure 5 shows solute-vacancy binding energy at up to sixth nearest-neighbor (6NN) separations within Al, Cu, and Ni hosts, where these are the energies to bind the solute and vacancy from effectively infinite separation. We see a large 1NN interaction in all three hosts, followed by mostly less than ±100 meV bindings for all other separations. We calculate the dissociation/association hop as between the 1NN and the 4NN. Therefore, we use the 4NN solute-vacancy binding energy as a measure of the term we have ignored. While it is not clear how to include these long-range binding effects rigorously in the full five-frequency model, we can qualitatively estimate their impact by correcting the energetics of the *ω*_3_ and *ω*_4_ hops so that they are consistent with the energy of complete dissociation. There are many ways to modify the dissociation/association hop barriers to ultimately obtain the correct long distance solute-vacancy binding. We choose to use the kinetically resolved activation (KRA) barrier approximation^29^, which divides the necessary 4NN correction energy in two and applies half to each of the *ω*_*3*_ and *ω*_*4*_ barriers. The new *ω*_*3*_ and *ω*_*4*_ hops are now reintroduced into the five-frequency model and all solute diffusivities are calculated again. Surprisingly we find that applying this solute-vacancy binding correction gives almost no change, and actually slightly worsens our comparison to experiment through the metric of ($E_{host}^{RMS}$, $E_{host}^{w-RMS}$, $D_{host}^{ratio}$). This shows that the effects of further neighbor solute-vacancy interactions do not have a significant effect on solute diffusivity compared to other sources of error in the systems we have tested, and we therefore assume it is of negligible importance for all the calculations in the present database. We note that some studies on BCC alloys have shown a potentially significant influence of these binding energies on some diffusion phenomena^30^.

**Figure 6:**
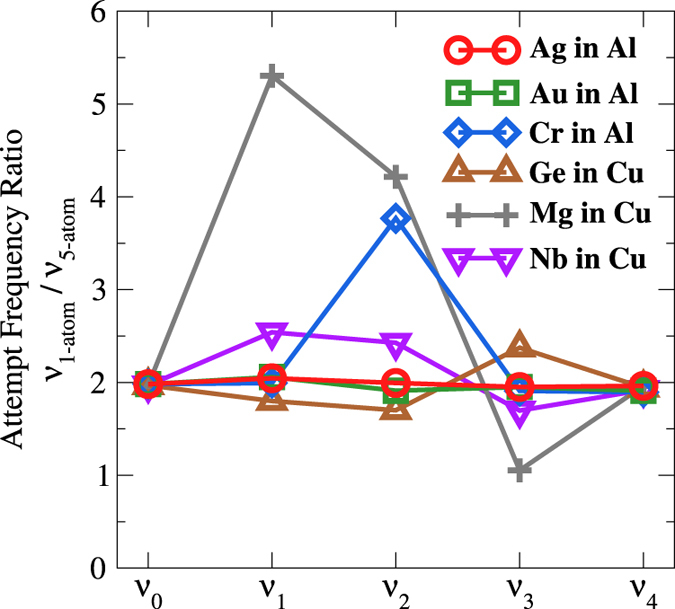
Calculated attempt frequency ratios for Ag, Au, and Cr in Al-host and Ge, Mg, and Nb in Cu-host. The ratio is between attempt frequencies between calculated using only the phonon vibrational modes of the migrating atom (*υ*_*1-atom*_) versus using the migrating atom and its four nearest atomic neighbors (*υ*_*5-atom*_). The attempt frequency for each of the five-frequencies is horizontally separated in the plot.

In calculating the attempt frequency prefactor for each jump in our diffusion model, we only considered the phonon modes of the migrating atom, as this produces a significant timesaving compared to including more atoms. While these modes capture a significant amount of information about changes in the attempt frequency, it assumes that the surrounding atomic phonon modes are not affected by the presence of the solute or vacancy. To assess the impact of the excluded modes, we extend the attempt frequency calculation to include 4 additional nearest atomic neighbor to the migrating atom. In Fig. 6, we plot, for several solutes in Al and Cu, the ratio between attempt frequencies calculated from only the migrating atom (*υ*_*1-atom*_) and from 4 additional atoms (*υ*_*5-atom*_). We see that by using additional phonon modes from surrounding atoms, the calculated attempt frequencies are generally reduced by a factor of about two for all frequencies. We see that there are some larger ratios for Mg and Cr, which for Mg may be because it is a light solute element, and for Cr the effect may be due to the spontaneous magnetic moment developed during the solute hop, *υ*_*2*_. To the extent that there is a uniform scaling of all attempt frequencies they will end up largely cancelling in the five-frequency model, leading to only the same scaling factor on the predicted diffusivity, with no change in the predicted diffusion activation barrier. Also, if *υ*_*0*_, the attempt frequency for the host self-hop, scales the same way as other hops, the accuracy of the predicted D values in this work would not be impacted by this shift as the prefactors are scaled by our DFT/experiment host self-diffusivity fitting correction scheme. For cases where the scaling is not constant the values appear to differ by at most a factor of 2.5 from the constant scaling of about 2, which will still lead to relatively small errors of at most about a factor of two. Therefore, we conclude that while phonon modes from additional neighboring atoms would produce a more accurate attempt frequency prefactor, it would not significantly improve solute diffusion predictions, particularly when our solute diffusion correction method is also being used.

## Usage Notes

We recommend direct usage of the reported solute diffusion coefficients, *D*_0_, and solute diffusion activation energy, *Q*, to generate temperature dependent solute diffusivities. Researchers who would like to instead regenerate the diffusivity data from the reported individual hop barriers and attempt frequencies should remember to apply the host self-diffusivity correction from Table 1. In other words, the difference between calculated solute diffusivity and the host self-diffusivity should be the quantity held in high confidence. We recommend caution when using the calculated diffusivity values of magnetic solutes, Cr, Mn, Fe, Co, and Ni in non-magnetic host alloys, as they exhibit much larger errors than other impurities when compared to experimental measurements.

## Additional Information

**How to cite this article**: Wu, H. *et al.* High-throughput ab-initio dilute solute diffusion database. *Sci. Data* 3:160054 doi: 10.1038/sdata.2016.54 (2016).

## Supplementary Material



## Figures and Tables

**Table 1:** Correctional shifts, *A*_*shift*_ and *E*_*shift*_, for DFT predicted Ag, Cu, Ni, Pd, Pt, and Mg self-diffusivity fitted from experimental diffusion

			**Uncorrected**		**Corrected**	
	**A**_**shift**_	**E**_**shift**_ **[eV]**	**D**_**0**_ **[cm**^**2**^ **/s]**	**Q [eV]**	**D**_**0**_ **[cm**^**2**^ **/s]**	**Q [eV]**
Al	12	0.20	0.00542	1.066	0.065	1.266
Cu	80	0.40	0.00353	1.680	0.282	2.080
Ni	500	0.47	0.00429	2.484	2.145	2.954
Pd	20	0.55	0.00360	2.096	0.072	2.646
Pt	20	0.85	0.00310	1.826	0.062	2.676
Mg	240	0.20	0.00568	1.206	1.362	1.406
The uncorrected and corrected DFT self-diffusion constant, *D*_*0*_, and activation barrier, *Q*, are also reported for each host element.						

**Table 2:** Predicted vacancy formation V_form_ (eV) and vacancy migration V_mig_ (eV) energies for the six host elements, Al, Cu, Ni, Pd, Pt, and Mg

	**Al**		**Cu**		**Ni**		**Pd**		**Pt**		**Mg**		**Average xc RMSE**	
	**V**_**form**_	**V**_**mig**_	**V**_**form**_	**V**_**mig**_	**V**_**form**_	**V**_**mig**_	**V**_**form**_	**V**_**mig**_	**V**_**form**_	**V**_**mig**_	**V**_**form**_	**V**_**mig**_	**V**_**form**_	**V**_**mig**_
PBE	0.485	0.581	0.963	0.717	1.645	0.957	1.137	0.959	0.611	1.215	0.798	0.408	0.432	0.110
LDA	0.580	0.603	1.269	0.830	1.587	1.078	1.407	1.127	0.878	1.425	0.802	0.415	0.264	0.082
PW91	0.461	0.538	1.025	0.698	1.333	0.930	1.113	0.916	0.608	1.169	1.196	0.396	0.509	0.138
PBEsol	0.632	0.606	1.249	0.805	1.580	1.059	1.363	1.084	0.840	1.393	0.831	0.413	0.287	0.069
Expt.^6^	0.67±0.03	0.61±0.03	1.28±0.05	0.70±0.02	1.79±0.05	1.04±0.04	1.70, 1.85	1.03±0.3	1.35±0.05	1.43±0.05	0.58–0.81	0.45–0.6	N/A	N/A
Different DFT exchange-correlation functionals, PBE, LDA, PW91, and PBEsol, are compared against experimental measurements. The average root mean squared error (RMSE) for each exchange-correlation functional has also been calculated for both V_form_ and V_mig_ separately. The migration energy for Mg is an average value of the basal and c-axis diffusivities.														
